# Numerical Demultiplexing of Color Image Sensor Measurements via Non-linear Random Forest Modeling

**DOI:** 10.1038/srep28665

**Published:** 2016-06-27

**Authors:** Jason Deglint, Farnoud Kazemzadeh, Daniel Cho, David A. Clausi, Alexander Wong

**Affiliations:** 1 Department of Systems Design Engineering, University of Waterloo, Ontario, N2L 3G1 Canada

## Abstract

The simultaneous capture of imaging data at multiple wavelengths across the electromagnetic spectrum is highly challenging, requiring complex and costly multispectral image devices. In this study, we investigate the feasibility of simultaneous multispectral imaging using conventional image sensors with color filter arrays via a novel comprehensive framework for numerical demultiplexing of the color image sensor measurements. A numerical forward model characterizing the formation of sensor measurements from light spectra hitting the sensor is constructed based on a comprehensive spectral characterization of the sensor. A numerical demultiplexer is then learned via non-linear random forest modeling based on the forward model. Given the learned numerical demultiplexer, one can then demultiplex simultaneously-acquired measurements made by the color image sensor into reflectance intensities at discrete selectable wavelengths, resulting in a higher resolution reflectance spectrum. Experimental results demonstrate the feasibility of such a method for the purpose of simultaneous multispectral imaging.

Multispectral imaging involves the capturing of imaging data of a particular scene or object at multiple wavelengths across the electromagnetic spectrum. Because different materials reflect, transmit, or emit at different wavelengths, multispectral imaging becomes a powerful tool to extract additional information about a scene or object. This approach facilitates unique material characterization and classification beyond what can be captured using conventional camera systems. As such, multispectral imaging has become a widely-used, powerful tool for different applications such as remote sensing^1^,^2^,^3^, material analysis^4^,^5^,^6^, and microscopy^7^,^8^,^9^,^10^.

Traditionally, multispectral imaging has often been performed in a sequential manner, where imaging data are captured at a specific wavelength in the electromagnetic spectrum. Such sequential multispectral imaging systems typically consists of a monochromatic sensor and a spectral filtering mechanism such as filter wheels^11^ and tunable filters^12^,^13^ that allow the desired wavelength of light to pass through for acquisition. While highly useful for imaging static phenomena in a controlled environment, there are several limitations associated with such sequential multispectral imaging systems. First, such imaging systems require a complex optical setup involving many optoelectronic elements, leading to a more expensive and less compact system. Second, the temporal resolution of such systems is reduced because the imaging is done in a sequential manner. This reduction in temporal resolution makes imaging dynamic or transient phenomena more challenging.

To address such limitations, there has been an on-going trend towards simultaneous multispectral imaging systems, where the goal is to capture imaging data at all desired wavelengths at the same time. Such systems allow for effective imaging of dynamic or transient phenomena. The traditional implementation of such devices tend to be complex and costly since numerous beamsplitting optics as well as dedicated imaging devices are required, proportional to the desired number of spectral channels. There has been a recent surge in design and development of on-chip multispectral imagers that facilitate a less complex, and more compact design for simultaneous multispectral imaging. A major limitation of such systems revolves around the use of customized multispectral image sensors, where different sets of pixels in the sensor array are configured to capture imaging data at a particular wavelength. For example, Park *et al*. propose a multispectral image sensor that is capable of capturing eight different wavelengths at the same time^14^. However, such custom multispectral image sensors are complex to manufacture and cost-prohibitive for many real-world applications. As such, a method for simultaneous multispectral imaging that leverages off-the-shelf, low-cost image sensors with standard color filter arrays (CFAs) is preferred.

There has been recent interest in exploring simultaneous multispectral imaging systems where higher spectral resolution is obtained from off-the-shelf image sensors with CFAs^15^,^16^,^17^,^18^,^19^,^20^,^21^. Such an approach to simultaneous multispectral imaging is based on the notion that the general characteristics of reflectance spectra of the samples being imaged are typical and more constrained in diversity, which is true for a wide range of specific applications. In essence, one can better leverage the measurements from off-the-shelf image sensors with CFAs, given the inherent characteristics of the imaging sensor and the imaging process, to infer higher resolution reflectance spectra. While this approach is not as flexible and reliable as spectroscopic approaches for obtaining a very wide diversity of reflectance spectra, it has been demonstrated to be effective for a wide range of applications such as artwork assessment^15^,^20^, clinical skin imaging^17^, vein visualization^22^, hemodynamic visualization^23^, and microscopy^9^. Such an approach is highly appealing in suitable scenarios as it greatly decreases the complexity, size, cost, and usability of multispectral imaging systems. In the most commonly-used configuration of this approach, simultaneous multispectral imaging, auto-correlation and cross-correlation are used to statistically model the relationship between the incoming light spectra and the image sensor measurements^15^,^16^,^17^,^18^,^19^,^20^. The resulting auto-correlation and cross-correlation models are then used to infer higher resolution reflectance spectra from sensor measurements via Wiener estimation. These inherent statistical assumptions limit the ability to predict the complex spectra behaviors and as such may not scale well for obtaining fine structure in higher resolution spectra such as absorption or emission lines.

In this study, inspired by our preliminary work on spectral inference^21^, we investigate the feasibility of performing simultaneous multispectral imaging using conventional sensors with Bayer CFAs. We leverage a comprehensive framework based on numerical demultiplexing of sensor measurements via spectral characterization of the image sensor and non-linear random forest modeling. By decoupling measurements that are captured using a low-cost, compact imaging system with conventional sensors with CFAs, one can predict higher resolution spectral data across multiple wavelengths within the sensitivity of the image sensor.

## Methods

The proposed numerical demultiplexing method can be summarized as follows. First, a comprehensive spectral characterization of the image sensor is performed. Second, given the spectral characterization information, a forward model is created that maps the input light spectra to the sensor measurements. Third, given the numerical forward model, the corresponding numerical demultiplexer is constructed via non-linear random forest modeling. This numerical demultiplexer can then be used to demultiplex simultaneously-acquired measurements made by the image sensor into reflectance intensities at discrete selectable wavelengths, resulting in a higher resolution reflectance spectrum. A more detailed description of each component of the proposed method is provided below.

### Imaging apparatus

In this study a Canon T3i APS-C CMOS image sensor was used to assess the feasibility of demultiplexing color image sensor measurements into higher spectral signals. The Canon T3i APS-C CMOS image sensor has a size of 22.3 mm × 14.9 mm and a Bayer pattern CFA, which we characterize in a comprehensive manner as described below.

### Spectral characterization

In order to construct a numerical forward model characterizing the formation of image sensor measurements given input light spectra, we must first quantitatively characterize the inherent spectral sensitivity of the image sensor in a comprehensive manner. The intensity measurement on a sensor’s pixel, *C*(*i*, *j*), can be described as


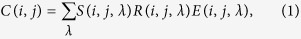


and depends on the spectral sensitivity of the sensor *S*(*i*, *j*, *λ*), the reflectance of the object *R*(*i*, *j*, *λ*), and the light source *E*(*i*, *j*, *λ*) which is used to illuminate the target^24^. Here (*i*, *j*) is the pixel location on the sensor and *λ* is the wavelength available at a given pixel. The spectral sensitivity, *S*(*i*, *j*, *λ*), can be changed by placing a CFA over the sensor, which facilitates the simultaneous acquisition of multiple spectral bands: red, green, and blue in the case of a Bayer CFA.

We characterize the spectral sensitivity of a sensor for a given color in the CFA by emitting a large set of discrete narrowband light spanning the desired wavelength range onto the sensor, and then record the corresponding spectral response within the range of wavelengths. For example, a very common CFA used in consumer-level color imaging systems is the Bayer pattern CFA^25^, which consists of red, green, and blue (RGB) filters placed on the sensor pixels resulting in three-channel spectral measurements. In this study, we designed and built a monochromator that enables wavelength selection in steps of 5 nm and we use the light emerging from the exit slit of the monochromator as an input into the camera to be imaged.

**Figure 1:**
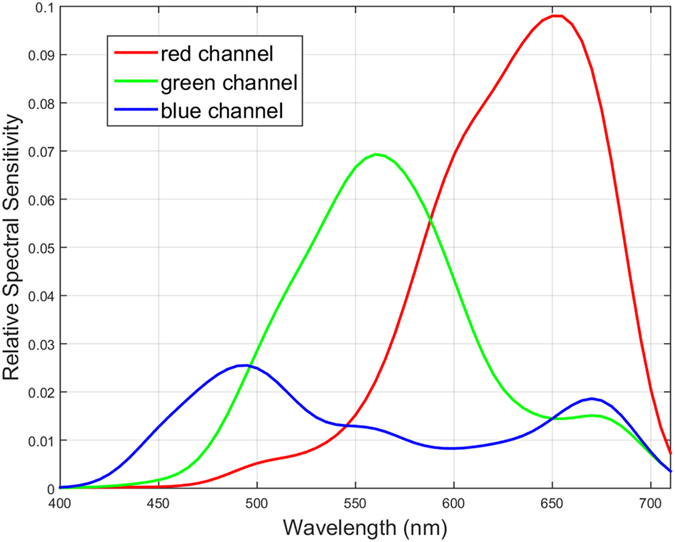
Spectral response of a Canon T3i APS-C CMOS image sensor with a Bayer pattern CFA. This spectral characterization is used to construct a forward model characterizing the formation of sensor measurements from light spectra hitting the sensor.

The focus of the camera is placed at infinity to ensure that the beam which impinges on the sensor is as close to collimation as possible, therefore uniformly illuminating a large region on the sensor which results in a large number of each of the three color filters. Using this setup we characterize the spectral sensitivity of a Canon T3i APS-C CMOS image sensor with a Bayer pattern CFA using a set of 61 discrete test spectra ranging from 410 nm to 710 nm. The spectral response curve for the three filters are shown in Fig. 1.

### Forward modeling

Given the spectral characterization of the color sensor, we can now construct a forward model characterizing the color measurement formation by the sensor with a CFA. By letting Λ(*i*, *j*, *λ*) = *R*(*i*, *j*, *λ*)*E*(*i*, *j*, *λ*), Equation 1 can be rewritten as


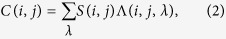


which can be written in matrix form as *C*_*p*×1_ = *S*_*p*×*n*_Λ_*n*×1_. Here *C* = [*c*_1_*c*_2_ ... *c*_*p*_]^*T*^ represents the measurements made by the image sensor using the *p* filter in the CFA, Λ = [*λ*_1_*λ*_2_ ... *λ*_*n*_]^*T*^ represents the intensities at *n* discrete selectable wavelengths of the light spectra arriving at the sensor, and *S* is the spectral sensitivity of the sensor. This relationship represents a forward model which maps the light spectra hitting the sensor to the sensor measurements made by the image sensor with a CFA.

### Numerical demultiplexer

At this stage, the goal is to construct a numerical demultiplexer based on the numerical forward model for the characterized image sensor described in Equation 2. One can treat the numerical demultiplexer as an inverse problem of the numerical forward model, with the goal of determining higher resolution reflectance spectra *Λ* given the image sensor measurements *C*:


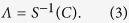


Here *S*^−1^(.) is an inverse function that outputs the higher resolution reflectance spectra *Λ* given the sensor measurements *C*. Given the complex relationship between the higher resolution reflectance spectra and the sensor measurements, and the fact that we have an under-determined system in this case, one cannot obtain the inverse function *S*^−1^(.) analytically. Therefore, in the proposed framework, we propose that a numerical demultiplexer can be constructed through nonlinear modeling of the relationship between reflectance spectra and sensor measurements.

We leverage non-linear random forest modeling^26^ to construct the numerical demultiplexer function *S*^−1^(.) using a comprehensive set consisting of 10,000 reflectance spectra and their corresponding sensor measurements based on the numerical forward model for the characterized image sensor. A random distribution of reflectance spectra was used to construct the numerical demultiplexer to ensure that all wavelengths are well represented ensuring that the numerical demultiplexer achieves strong demultiplexing performance across the entire range of wavelengths. The nonlinear random forest model, used in this study for constructing the numerical demultiplexer, is comprised of 8,000 decision trees in total.

A key advantage of using such a non-linear random forest modeling approach to constructing the numerical demultiplexer is that it allows for reliable and flexible modeling of the complex relationships between reflectance spectra and sensor measurements without imposing strong assumptions about the nature of the relationship. Furthermore, we also introduce a numerical Wiener-based demultiplexer based on auto-correlation and cross-correlation models for comparison purposes with the proposed random forest-based demultiplexer. The Wiener-based demultiplexer based model is learned using the numerical forward model for the characterized image sensor described in Equation 2.

Given the constructed numerical demultiplexer, one can demultiplex simultaneously-acquired sensor measurements made by the characterized image sensor with a CFA into higher resolution reflectance spectra.

### Quantitative performance assessment

**Figure 2:**
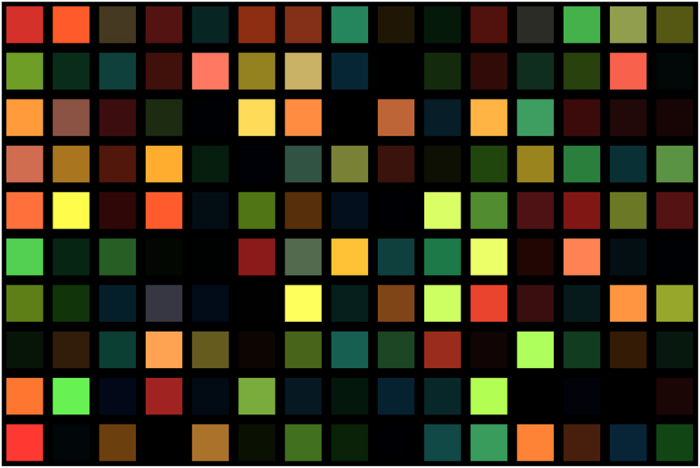
A random selection of 150 test sensor measurements (shown here as patches in a chart) from the set of 10000 test sensor measurements.

We performed two different sets of experiments to assess the feasibility of the proposed framework. In the first set of experiments, we performed a quantitative performance assessment of the proposed framework within a controlled simulation environment. More specifically, a simulated sensor was constructed based on the characterization of the Canon T3i sensor with a Bayer pattern CFA, and a total of 10,000 new randomized simulated reflectance spectra were then generated and captured using the simulated sensor to generate sensor measurements. A random selection from the set of 10,000 test sensor measurements used in the first set of experiments is shown in Fig. 2. These RGB measurements are then fed into the numerical demultiplexer to obtain predicted reflectance spectra. The predicted reflectance spectra are then compared quantitatively against the original reflectance spectra entering the sensor using the peak signal-to-noise ratio (PSNR) to assess the fidelity of the demultiplexed spectra.

**Figure 3:**
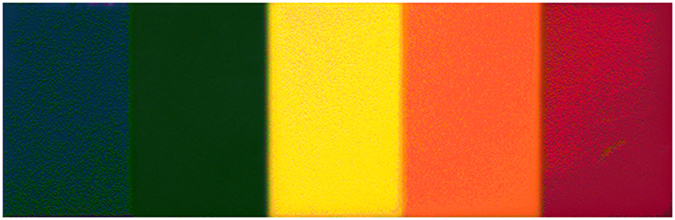
The test icon used in the second set of experiments. The true reflectance spectrum was measured for each unique section of the icon and then compared to the predicted spectra from the three inverse methods.

In the second set of experiments, we wish to validate the observations made from the first set of controlled simulation experiments within a real-world setting. To accomplish this we used the real spectrally-characterized Canon T3i sensor with a Bayer pattern CFA to capture measurements of a test icon (see Fig. 3). The reflectance spectra of each section in the test icon was determined by measuring the sections using a high-resolution spectrometer while being illuminated by a Halogen-Tungsten (2650 k) broadband light source under a 45°–0° receiver-source setup. The true reflectance spectra were then found by detrending the measured spectra by the reflectance spectrum of the light source using a 99% reflectance target.

The sensor measurements were then fed into the numerical demultiplexer to obtain predicted reflectance spectra. The demultiplexed reflectance spectra from the numerical demultiplexer were then compared quantitatively against the known reflectance spectra of the icon using PSNR to assess the fidelity of the demutiplexed spectra.

**Figure 4:**
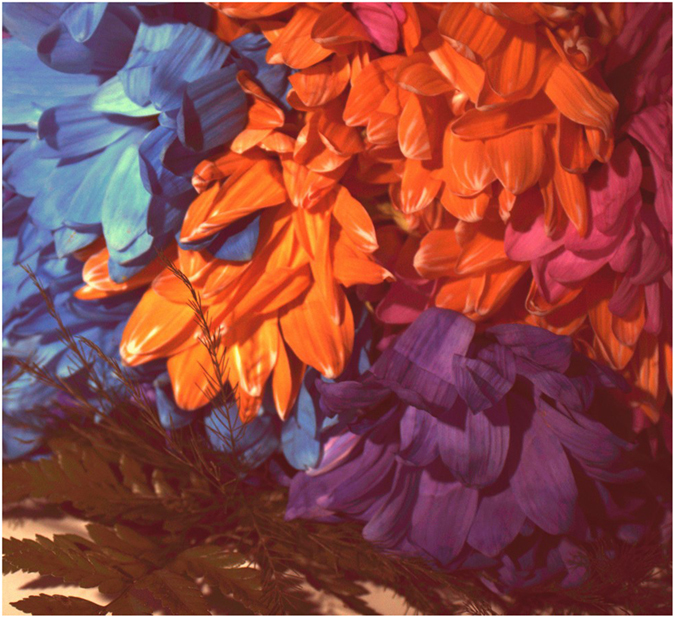
Sensor measurements of a scene consisting of flowers of different colors using real spectrally-characterized Canon T3i sensor with a Bayer pattern CFA used in the second set of experiments.

Finally, we use the real spectrally-characterized Canon T3i sensor with a Bayer pattern CFA to capture measurements of a scene consisting of flowers of different colors to qualitatively illustrate the feasibility of the proposed framework (see Fig. 4). The sensor measurements were then fed into the numerical demultiplexer to construct predicted reflectance spectra images at different spectral wavelengths. Three specific spectral wavelengths (490 nm, 550 nm and 610 nm) were chosen for illustrative purposes to highlight key differences between the tested methods.

As a baseline, a state-of-the-art and popular Wiener Estimation (WEM) method^16^,^17^,^22^ was also evaluated alongside the proposed Wiener-based demultiplexer (DEMUX-WEM) and random forest-based demultiplexer (DEMUX-RFM), using the same procedures for both sets of experiments.

## Results

The goal of this study is to investigate the feasibility of simultaneous multispectral imaging with CFAs via numerical demultiplexing of sensor measurements. The experimental results from the two sets of experiments for assessing the feasibility of this proposed approach are presented below.

**Figure 5:**
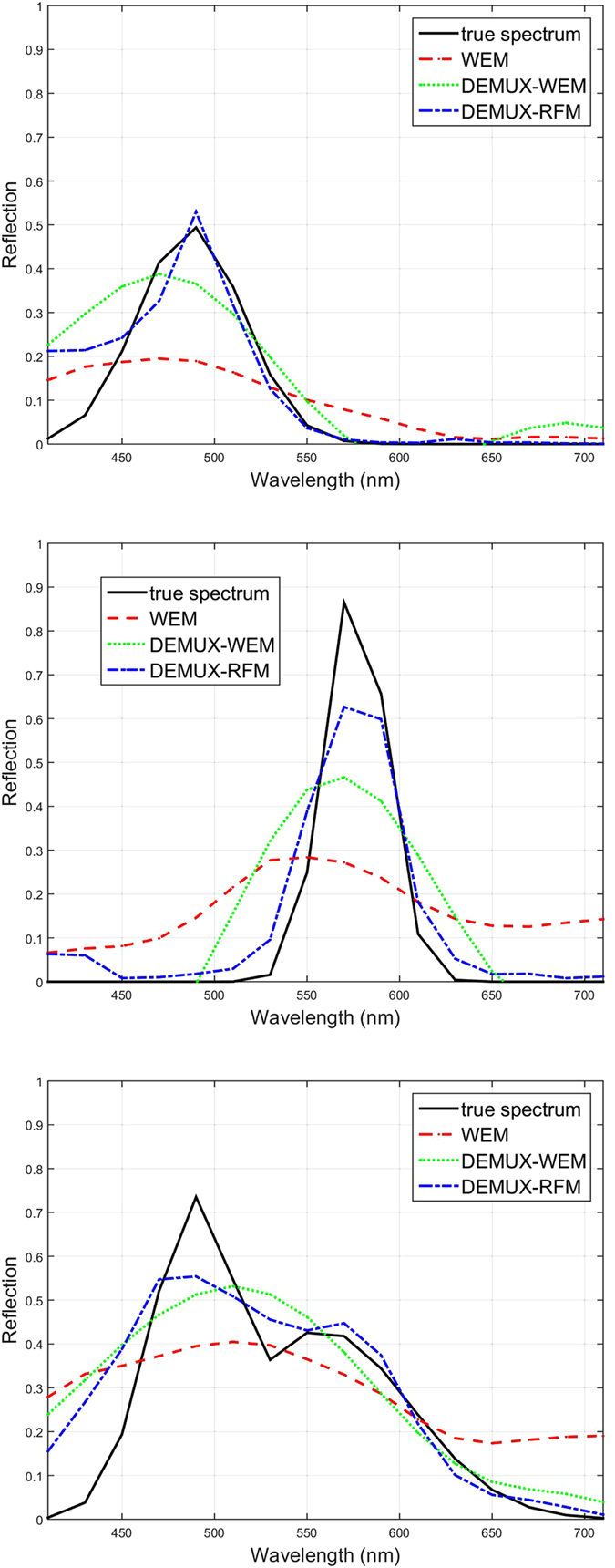
Three examples of simulated light spectra that were captured using the simulated sensor to obtain image sensor measurements (see Fig. 2). These sensor measurements were then used by the three tested approaches to predict higher resolution reflectance spectra. The proposed random forest-based demultiplexer (DEMUX-RFM) outperformed both Wiener estimation method (WEM) and proposed Wiener-based demultiplexer (DEMUX-WEM).

In the first set of experiments, we wish to perform a comprehensive performance assessment of the proposed framework within a controlled simulation environment. The PSNR of WEM, DEMUX-WEM, and DEMUX-RFM for the first set of experiments were 14.7 dB, 17.8 dB and 20.16 dB, respectively. The proposed DEMUX-WEM achieved a significant PSNR improvement over the traditional WEM, with the proposed DEMUX-RFM exhibiting significant PSNR improvements over the other two methods. This illustrates that the efficacy of the proposed DEMUX-WEM and DEMUX-RFM are providing more generalizable approaches for predicting a greater diversity of reflectance spectra. Three test reflectance spectra are shown in Fig. 5, along with the predicted spectra from the tested methods. The top two spectra exhibit the prediction accuracy with a unimodal shape while the bottom spectra exhibits the prediction accuracy with a bimodal shape. In all three cases, the proposed DEMUX-RFM provided the most accurate predicted spectra, followed by DEMUX-WEM and then WEM.

In the second set of experiments, we wish to validate the observations made regarding the proposed framework in the first set of controlled simulation experiments within a real-world environment. To accomplish this, we used the real Canon T3i sensor with a Bayer pattern CFA to capture measurements of a test icon (as seen in Fig. 3), and of a scene consisting of flowers of different colors.

The PSNR of WEM, DEMUX-WEM, and DEMUX-RFM for the second set of experiments involving the test icon are 17.7 dB, 13.3 dB, and 17.2 dB, respectively. While WEM achieves the highest PSNR in this set of experiments, it is important to note that the primary reason why WEM is able to achieve this level of performance is that the true reflectance spectra of the sections in the test icon very closely resembles the spectra of color patches in the Macbeth chart, which are used to train WEM as per^16^. Nevertheless, it is very interesting to observe that the proposed DEMUX-RFM, which is constructed based on the forward model of the characterized sensor, is able to achieve a PSNR that is very close to the WEM PSNR, with a difference of just 0.5 dB, which illustrates the strength of the proposed framework.

**Figure 6:**
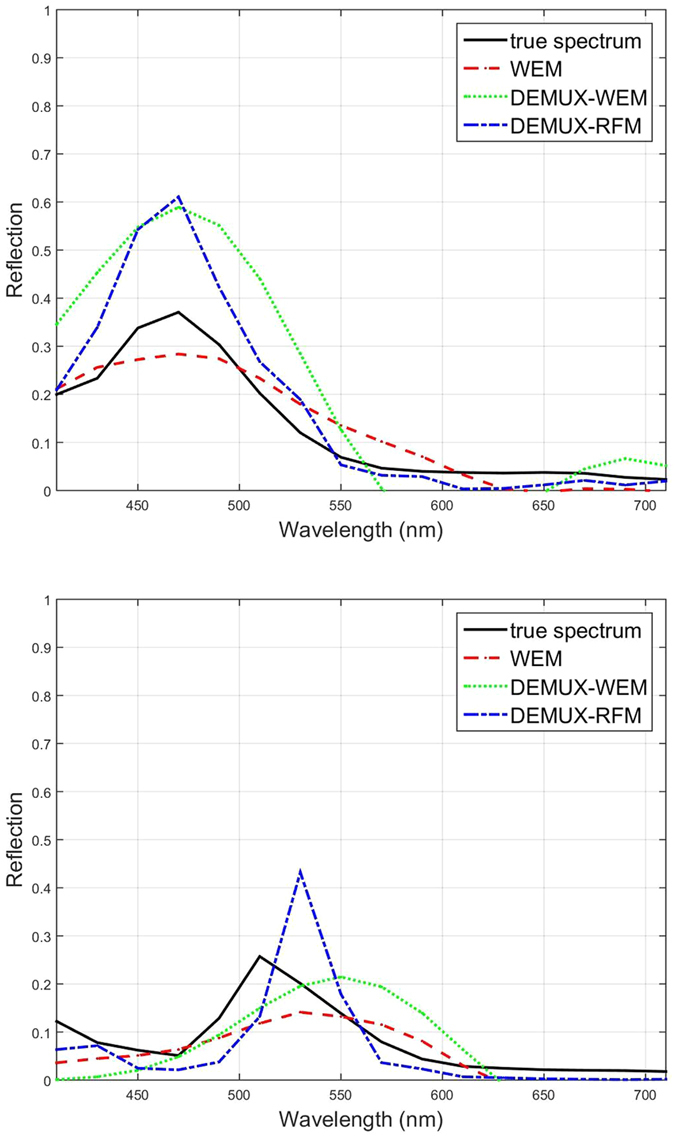
Two of the five true reflectance spectra (‘blue’ and ‘green’) from the test icon (see Fig. 3), along with the corresponding predicted spectra obtained from a state-of-the-art Wiener Estimation Method (WEM), the proposed Wiener-based demultiplexer (DEMUX-WEM) and the random forest-based demultiplexer (DEMUX-RFM). Top: The ‘blue’ true spectrum and the predicted spectra produced using the inverse methods have similarly shaped spectral curves. Bottom: the predicted spectra from DEMUX-RFM is closest to the true ‘green’ spectrum.

The true spectra and predicted spectra from the test methods for the ‘blue’ and ‘green’ sections of the test icon are shown in Fig. 6. When predicting the spectrum of the ‘blue’ section, all three methods exhibited similar performance. However, when predicting the spectrum of the ‘green’ section, WEM and DEMUX-WEM exhibited similar performance while DEMUX-RFM achieved a more accurate prediction.

**Figure 7:**
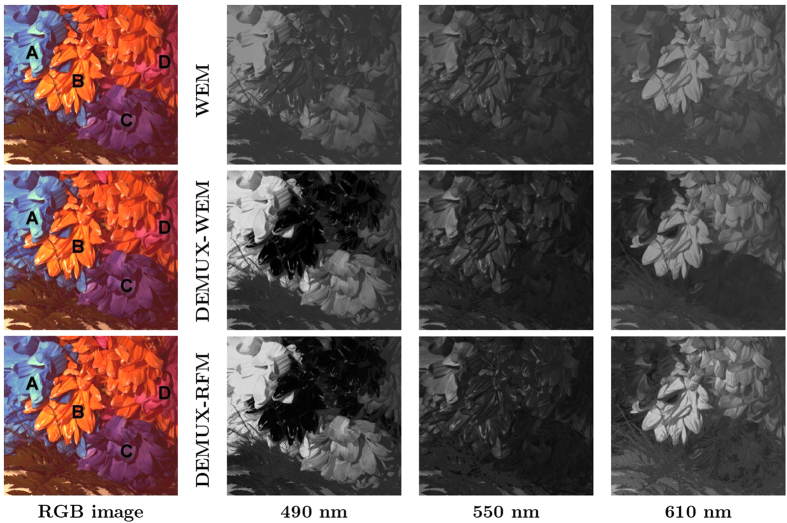
Predicted reflectance spectra images at three specific spectral wavelengths (490 nm, 550 nm and 610 nm) for the scene of flowers with different colors used in the second set of experiments. The three specific wavelengths are chosen for illustrative purposes to highlight key differences between the tested methods.

The predicted spectral images at three different spectral wavelengths (490 nm, 550 nm and 610 nm) were obtained using the WEM, DEMUX-WEM, and DEMUX-RFM methods for a scene consisting of flowers with different colors as shown in Fig. 7. A number of interesting observations can be made from the predicted spectral images. It can be observed that Flower A can be seen to have noticeably higher intensities at 490 nm for the DEMUX-WEM and DEMUX-RFM methods, particularly with higher intensity contrast between Flower A and the other flowers, when compared to the WEM method. Since Flower A is a blue flower it has a significantly higher reflectance at 490 nm compared to the other flowers. This illustrates the efficacy of the proposed DEMUX-WEM and DEMUX-RFM methods in this case.

It can also be observed that Flower B has a very low intensity at 490 nm for the DEMUX-WEM and DEMUX-RFM methods, while the WEM method shows noticeably higher relative intensity at 490 nm in comparison. Given that Flower B is an orange flower and has very low reflectance at 490 nm, the predicted reflectance spectra produced by the DEMUX-WEM and DEMUX-RFM methods more accurately represent the scene compared to the WEM method. Furthermore, it is interesting that there is noticeably higher contrast between Flower B and Flower D at both 490 nm and 610 nm with the proposed DEMUX-WEM and DEMUX-RFM methods compared to the WEM method. In particular, Flower B and Flower D are visually inseparable from the spectral image produced using WEM at 610 nm. In addition, Flower B is orange and exhibits higher reflectance than Flower D at 610 nm, since 610 nm corresponds to the orange portion of the visible band. These improved reflectance spectra predictions illustrate the efficacy of the proposed DEMUX-WEM and DEMUX-RFM methods.

It can be further observed that Flower B has a noticeably higher intensity at 610 nm for the DEMUX-RFM method when compared to both DEMUX-WEM and WEM, with noticeably greater contrast between Flower B and other flowers at 610 nm when compared to DEMUX-WEM and WEM. Given that Flower B is an orange flower and exhibits higher reflectance at 610 nm (orange color in the visible spectrum), the superior performance of the proposed DEMUX-RFM method is demonstrated compared to the other methods.

Finally, Flower C has noticeably higher intensity at 610 nm for DEMUX-RFM compared to DEMUX-WEM, and noticeably lower intensity at 550 nm for DEMUX-RFM compared to WEM. Given that Flower C is a purple flower and is characterized by high reflectance at 490 nm, very low reflectance at 550 nm, and mild reflectance at 610 nm, the results of DEMUX-RFM are more representative of the reflectance spectra of Flower C compared to DEMUX-WEM and WEM.

## Discussion

In this study we assess the feasibility of simultaneous multispectral imaging using conventional image sensors with color filter arrays. We accomplish this by leveraging a comprehensive framework for numerical demultiplexing of the color image sensor measurements via non-linear random forest modeling.

Comprehensive quantitative performance assessment within a controlled simulation environment as well as with real-world measurements of a test icon demonstrate the efficacy of the proposed framework. The experimental results demonstrate that such an approach can be used to enable simultaneous multispectral imaging using conventional image sensors with standard CFAs. The numerical demultiplexing method works in scenarios when the reflectance spectra of the samples being imaged are typical and constrained in both diversity and anomalous peculiarities, which is true for a wide range of specific applications.

The noticeable PSNR gains achieved while using the proposed framework may stem from the fact that a comprehensive spectral characterization of the detector was introduced to obtain an accurate computational forward model, which was then used to build a numerical demultiplexer. This differs from the previously proposed Wiener estimation method which aims to learn a parametric statistical model that maps sensor measurements to input light spectra directly using spectral characterization information. By first building a more accurate computational forward model, which is based on the spectral characterization of the image sensor, a more accurate numerical demultiplexer can be constructed. This numerical demultiplexer can in turn predict a greater variety of reflectance spectra in a more reliable manner. This factor is reinforced by the first experimental results, where both of the proposed numerical demultiplexers (DEMUX-WEM and DEMUX-RFM) performed noticeably better than the Wiener estimation method (WEM) when tasked to predict a wide variety of reflectance spectra.

A key factor to the noticeable PSNR gains achieved using the proposed method (DEMUX-RFM) when compared to the alternate proposed method (DEMUX-WEM) may stem from the fact that a non-linear random forest model was used to construct the numerical demultiplexer. This non-linear inverse model is less prone to overfitting and more flexible than the parametric statistical model used in Wiener-based methods. This allows for a more robust and generalized demultiplexer that performs well for predicting a greater diversity of reflectance spectra and is less sensitive to illumination variations. This factor is reinforced by the experimental results from both sets of experiments, which showed that the proposed random forest-based numerical demultiplexer (DEMUX-RFM) performs noticeably better than the proposed Wiener-based numerical demultiplexer (DEMUX-WEM).

As we have demonstrated in this study, demultiplexing of RGB color sensor measurements into higher spectral signals is possible via a numerical demultiplexing. This simultaneous multispectral imaging method has strong implications for many applications where low-cost, low-complexity, and portable simultaneous multispectral imaging systems are highly desired. Future work, extending the current research, involves investigating alternative models for constructing the numerical demultiplexer and the integration of a more comprehensive forward model.

## Additional Information

**How to cite this article**: Deglint, J. *et al*. Numerical Demultiplexing of Color Image Sensor Measurements via Non-linear Random Forest Modeling. *Sci. Rep*. **6**, 28665; doi: 10.1038/srep28665 (2016).
